# Evaluation of subsurface transport processes of delayed gas signatures applicable to underground nuclear explosions

**DOI:** 10.1038/s41598-022-16918-5

**Published:** 2022-08-01

**Authors:** Charles R. Carrigan, Yunwei Sun, Tarabay Antoun

**Affiliations:** Lawrence Livermore National Laboratory, Livermore, CA USA

**Keywords:** Environmental sciences, Hydrology

## Abstract

Radioactive gas signatures from underground nuclear explosions (UNEs) result from gas-migration processes occurring in the subsurface. The processes considered in this study either drive or retard upward migration of gases from the detonation cavity. The relative importance of these processes is evaluated by simulating subsurface transport in a dual-permeability medium for the multi-tracer Noble Gas Migration Experiment (NGME) originally intended to study some aspects of transport from a UNE. For this experiment, relevant driving processes include weak two-phase convection driven by the geothermal gradient, over pressuring of the detonation cavity, and barometric pumping while gas sorption, dissolution, radioactive decay, and usually diffusion represent retarding processes. From deterministic simulations we found that over-pressuring of the post-detonation chimney coupled with barometric pumping produced a synergistic effect amplifying the tracer-gas reaching the surface. Bounding simulations indicated that the sorption and dissolution of gases, tending to retard transport, were much smaller than anticipated by earlier laboratory studies. The NGME observations themselves show that differences in gas diffusivity have a larger effect on influencing upward transport than do the combined effects of tracer-gas sorption and dissolution, which is consistent with a Sobol’ sensitivity analysis. Both deterministic simulations and those considering parametric uncertainties of transport-related properties predict that the excess in concentration of SF$_6$ compared to $^{127}$Xe as might be captured in small volumetric samples should be much smaller than the order-of-magnitude contrast found in the large-volume gas samples taken at the site. While extraction of large-volume subsurface gas samples is shown to be capable of distorting in situ gas compositions, the highly variable injection rate of SF$_6$ into the detonation cavity relative to that of $^{127}$Xe at the start of the field experiment is the most likely explanation for the large difference in observed concentrations.

## Introduction

Detectable gas releases from underground nuclear explosions (UNEs) have typically been characterized as being either prompt or delayed. Prompt venting occurs as a result of a nearly immediate, major failure of a containment zone surrounding the detonation cavity producing a rapid and usually focused escape of gases from the detonation cavity. In contrast, delayed releases may not give rise to detectable signatures for days to months following a UNE. Test site operations, such as opening tunnels or drilling back into the detonation cavity, as hypothesized for the appearance of atmospheric gases from the DPRK 2013 UNE almost two months after the detonation^1,2^, represent one type of delayed release. The other type, which is the subject of this study, is a result of the gradual, primarily fracture-network-based migration of gases from the detonation cavity across an otherwise competent containment zone and released over a broad area at the surface^1,3,4^. Depending among other things on the geology of a UNE site, depth of detonation, nuclear yield, and atmospheric pressure record, it was predicted such delayed releases at the surface could potentially produce detectable atmospheric signatures at distances of tens to more than a thousand kilometers given current radioxenon analysis sensitivities^5^. Besides representing a possible mode of detectable radioxenon release in addition to prompt venting for the atmospheric identification of UNEs, the potential for local atmospheric monitoring of broad-area and delayed signatures also suggests its use as a tool for more efficiently locating the detonation site during an on-site inspection carried out as proposed by the Comprehensive Nuclear Test-Ban Treaty (CTBT).

Different combinations of physical transport processes relevant to delayed signatures (e.g., barometric pumping and dissolution of gases in groundwater in a fractured regime) have been studied at different levels of detail (e.g.,^3,5–7,9,10, 27–31^) to evaluate the effects on migration of noble gases from a UNE. However, UNE gas migration processes are complex, interdependent, and temporally and spatially dependent^6,11,12^. Increasing model fidelity of noble gas transport in complex and uncertain geological systems typically requires a large increase in computational and experimental resources necessary for model development, model validation, and parameter calibration. Thus, model complexity is often reduced in conceptualizing a UNE by focusing only on certain physical processes to the exclusion of others. This raises the question regarding how much neglected physical processes impact predictions of noble gas signatures relevant to UNE detection, and if the tradeoff that is often made between model fidelity and computational expense is justified.

Field experiments involving the release and monitoring of gases are a necessary component for understanding what physical processes and material properties are most important for influencing gas migration from the post-detonation cavity and for testing models of gas transport. In turn, adequately validated models of gas transport offer the potential to generalize the results of a field experiment carried out at a particular site to other hydrogeologic environments that may also characterize UNE sites. The first published tracer experiment performed at two historic UNE test sites on Rainier Mesa at the Nevada Test Site (now NNSS – Nevada National Security Site) used the inert chemical tracers sulfur hexafluoride (SF$_6$) and Freon 13B1 to evaluate aspects of the natural release of radioactive effluents from past UNEs^13^. A significant finding of the experiment was that detonation-cavity pressurization was more likely to induce subsurface horizontal transport along high-permeability rock layers while atmospherically induced transport or barometric pumping is more efficient for drawing gases vertically to the surface. Another field experiment, known as the Non-Proliferation Experiment (NPE), focused on delayed release and detection of gases from a buried one-kiloton chemical explosion beneath Rainier Mesa at NNSS. SF$_6$ and $^3$He tracers were released simultaneously into the detonation cavity by the explosion and gradually migrated across the 400-meter zone of containment^3^. Soil gases were monitored for both tracers at many locations on the surface over a period of 500 days. The experiment showed that strong chromatographic effects should be expected during migration of gases in fractures with the high atomic mass, low-diffusivity SF$_6$ tracer arriving at the surface months before the low atomic mass, high-diffusivity $^3$He tracer when barometric pumping^4^ was the dominant mode of transport. A computer model of the transport regime matching the observed arrival times of both tracers was then developed. The calibrated model, which included gas dissolution in groundwater represented by Henry’s law, was then used to predict that both the delayed $^{133}$Xe and $^{37}$Ar gas signatures, with half-lives of 5.24 and 34.95 days respectively, should be detectable at the surface during periods of decreasing atmospheric pressure before the signals were lost to radioactive decay. Additionally, it was demonstrated that the interconnection between explosion-produced fracturing and the pre-existing natural fault and fracture system on Rainier Mesa provided pathways for gas migration to the surface. As part of the Underground Nuclear Explosion Signatures Experiment (UNESE), two different field experiments were carried out starting in 2012 at the Barnwell historic UNE site on Pahute Mesa at NNSS. In the first experiment, Freon 12B1 tracer was injected into the detonation zone consisting of rubblized rock. Weak pressurization ($\sim$ 40 mb) was maintained in this zone for a period of 10 days to simulate post-detonation conditions^1^. Tracer was detected on the surface after 2 days with subsequent concentration peaks during periods of falling atmospheric pressure that were approximately one-hundred times greater than peaks occurring days after the period of pressurization which supports modeling presented in this study on enhanced surface concentrations resulting from weak cavity pressurization combined with barometric pumping. It was also found with the aid of near real-time monitoring that, using the newly developed LLNL Subsurface Gas Smart Samplers, radon levels in soil gas samples were 10–15 times greater during weak pressurization of the cavity as might occur following a UNE. This was the first study to propose the use of radon as a natural tracer for rapid soil-gas surveys to isolate localized areas of subsurface residual pressurization due to a possible clandestine UNE test during an on-site inspection (see^14^ for additional discussion). The second experiment, also carried out at the Barnwell site in 2013^15^, involved both radioactive and chemical tracers which allows comparison with some of the results of this paper and is reserved for later discussion in this section.

More recently, Freon 12B2 was injected into the historic Disko Elm rubble-filled cavity beneath Aqueduct Mesa at NNSS also as part of UNESE^16^. The Aqueduct Mesa zone of containment is about 1/10 as permeable as that of the Barnwell site. Even so, tracer was detected on the surface exhibiting concentration amplitudes that were closely matched using a multi-parameter variational analysis constrained by the actual cavity and surface pressure histories. It was observed that tracer was only detected on the Mesa during periods when the altitude-corrected surface pressure fell below the cavity pressure. In the studies summarized here, chemical tracers were used to evaluate the response of subsurface gases subjected to different combinations of forcing under a range of hydrogeologic conditions. Additionally, when time-dependent surface and cavity pressure observations were matched with computer simulations, the output yielded estimated parameters (e.g., fracture aperture, fracture density, and fracture permeability) relevant to the transport of gases in general.

In the previous studies chemically inert tracers such as SF$_6$ or Freon were preferred for a variety of reasons to using the actual radionuclide gases (e.g., $^{[131\text{m }~133\text{m }~133~135]}$Xe, and $^{37}$Ar) themselves. Inert chemical tracers are extremely attractive since their cost is quite low to acquire, transport, inject, sample, and analyze compared to the radionuclide tracers. However, it is also a legitimate question to ask how well chemical tracers such as SF$_6$ or Freon can be expected to serve as replacements for the radionuclides of interest. This question was partially addressed recently in a novel study by Stroujkova et al.^17^. The authors conducted a field experiment involving injection of equal volumes of SF$_6$ and stable Xe into an existing shallow cavity and fracture zone formed by a small detonation in granitic rock with a shallow water table. Concentrations of SF$_6$ tracked those of Xe typically within a factor of 2 over an observed range of 10$^6$ with the relatively small differences explained by differences in diffusivity and dissolution properties of the two gases.

The sample volume required to analyze a particular tracer highlights another important difference between using inert chemical tracers and radioxenon isotopes. Individual gas samples taken in all the chemical-tracer studies mentioned above had volumes typically between 0.05–0.5 liters while radioactive tracer samples obtained in the second Barnwell site experiment^15^ required sample volumes of about 2 cubic meters (2000 liters). Extracting such large samples from a very low-volume fracture system raises significant questions about the degree that sampling can interfere with observing the transport processes that we want to understand. Additionally, we will also consider in the Discussion and Conclusions section how acquiring such large samples from a low-volume fracture-dominated system might distort the chemical or isotopic signatures that we want to measure.

**Figure 1:**
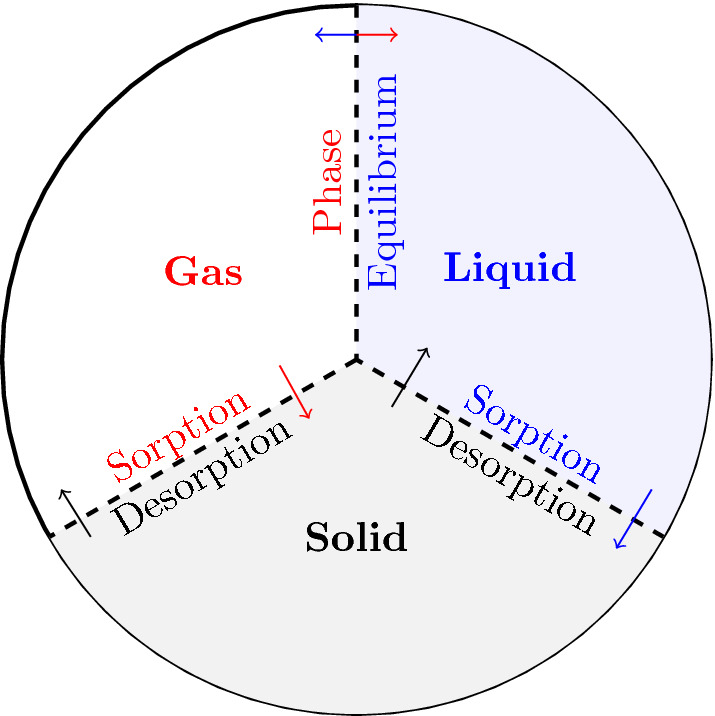
Schematic of the representative elementary volume (REV) conceptualized in the simulations. The volume fraction of liquid and gas phases denotes the porosity while the volume ratio of liquid phase to the porosity is defined as saturation. The mass exchange between gas and solid phases is described by sorption/desorption and that between gas and liquid phases is described by phase equilibrium.

The second Barnwell site experiment alluded to earlier, the Noble Gas Migration Experiment (NGME^15^), potentially allowed making comparisons of tracer migration involving the transport of SF$_6$ along with the two radioactive tracers, $^{127}$Xe and $^{37}$Ar. Details of the penetration of the U-20az borehole into the Barnwell detonation cavity are given in Carrigan et al.^1^, Fig. 1a and , Fig. 1) and a detailed description of the NGME experimental approach is given by Olsen et al.^15^. $^{37}$Ar and $^{127}$Xe were pre-mixed and then injected contemporaneously with but separately from SF$_6$ into the U-20az cavity over a 10-hour period. Injection flow rates for the pre-mixed Ar/Xe tracers and the SF$_6$ tracer were periodically monitored and adjusted separately by manually changing tracer injection rates. Ninety-nine days following the injection of the tracers, the cavity was pressurized for 46 hours by injecting air at $\sim$23 m$^3$min$^{-1}$. Gas samples were collected at or just below ground surface and analyzed for the above mentioned three gases.

Barometric pumping has been extensively modeled in fractured rock as the late-time driving force of gas transport from UNEs (e.g.,^3,4,7,8,18,19^). We will use the atmospheric-pressure-fluctuation history at the Barnwell site covering the period of the NGME experiment as well as over-pressuring of the chimney initiated 99 days after injection of the three tracers. Gas sorption in crushed rock powder and intact core samples has been modeled as an interaction between gas and solid phases in a batch mode under dry conditions using Henry’s type and Langmuir isotherm models^20,21^. Recently, Neil et al.^22^ studied the combined effect of gas sorption and dissolution in partially saturated zeolites. It should be noted that our models include the well-documented groundwater saturation distribution at the Barnwell site which evolves following the formation of the collapsed cavity. While our simulations include the effects of partial saturation on reducing adsorption of xenon in the zeolitic containment zone, they would still appear to overestimate the adsorption of xenon compared to that observed by Neil et al.^22^ in their laboratory experiments. Solubilities of argon and xenon were determined as functions of temperature and pressure^17–24^ using experimental data under saturated conditions. Experimental data on SF$_6$ dissolution (between gas and liquid phases) are also available for several temperature ranges^25–28^. Since those calibrated sorption and dissolution models in batch mode have not been coupled with single-gas phase or multiphase transport, the retarding role of sorption and dissolution has not been previously quantified but will be considered further here.

In addition to the base-case processes involving two-phase convection due to the natural geothermal temperature gradient (i.e., 25 $^{\circ }$C km$^{-1}$) and gas diffusion, we consider the impact of four physical processes: gas sorption, dissolution, overpressure created by air injection into the Barnwell cavity, and barometric pumping, on gas signals at the ground surface of the NGME system. We first simulate physical processes calibrated using sorption isotherms and dissolution models from literature data. We also conduct both an uncertainty quantification and sensitivity analysis of those four physical processes using PSUADE^29^. The result of a Sobol’ sensitivity analysis^30^ provides a quantitative measure for determining the relative influences of the different processes on gas transport as well as the tradeoff between model fidelity and computational expense.

## Materials and methods

In this section, we describe physical processes that can affect gas signatures from UNEs. Nonisothermal multiphase reactive transport in a fractured rock system has been modeled for water, air, and gas components in liquid, gas, and nondeformable solid phases^12^ using the NUFT program^31,32^. Water and air are the major components in the liquid and gas phases, while other gas components are minor components with relatively low mass fractions in these two phases. To distinguish gas sorption (interaction between gas and solid) from gas dissolution (phase equilibrium between gas and liquid), the laboratory experiments involving gas sorption on a mineral surface are typically conducted in the absence of water saturation. On the other hand, gas dissolution behavior is determined in the absence of a solid phase. Because field experiments do not monitor gas migration at levels adequate to capture the dynamics of gas sorption on solids and dissolution into the liquid phase, we use laboratory experimental data obtained under idealized conditions tending to maximize adsorption effects to evaluate the contribution to transport of sorption and dissolution separately and in combination. As shown in Fig. 1, the gas sorption is often described by a linear (or Henry’s type) isotherm and Langmuir kinetics (or isotherm) while the phase equilibrium is represented using various dissolution models including Henry’s law equilibrium model. It should be noted that Henry’s Law in its original form describes the equilibrium concentrations of a gas component existing between the gas phase and a dissolved phase in the water. However, the linear function of Henry’s Law is also used to describe Henry’s-law-type absorption isotherm appropriate for low gas concentrations.

### Gas sorption isotherms

**Figure 2:**
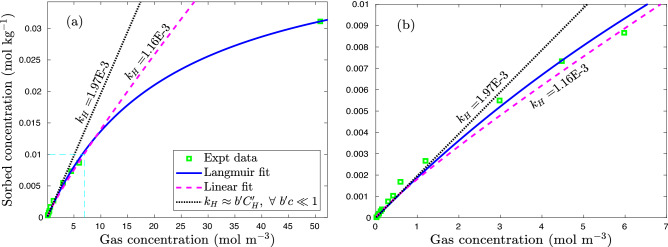
Comparison between Henry’s type and Langmuir isotherms of xenon sorption in shale at 0 $^{\circ }$C. (**a**) Langmuir isotherm fits well with experimental data in the full concentration range ($0 \le c \le 55$ [mol m$^{-3}$]). (**b**) Henry’s type isotherm approximates experimental data in the low concentration range ($0 \le c \le 7$ [mol m$^{-3}$]). The solid curve (blue) represents the Langmuir fit while dashed (magenta) and dotted (black) lines are the Henry’s type fit and the linearized Langmuir fit when $b'~c\ll 1.$

**Figure 3:**
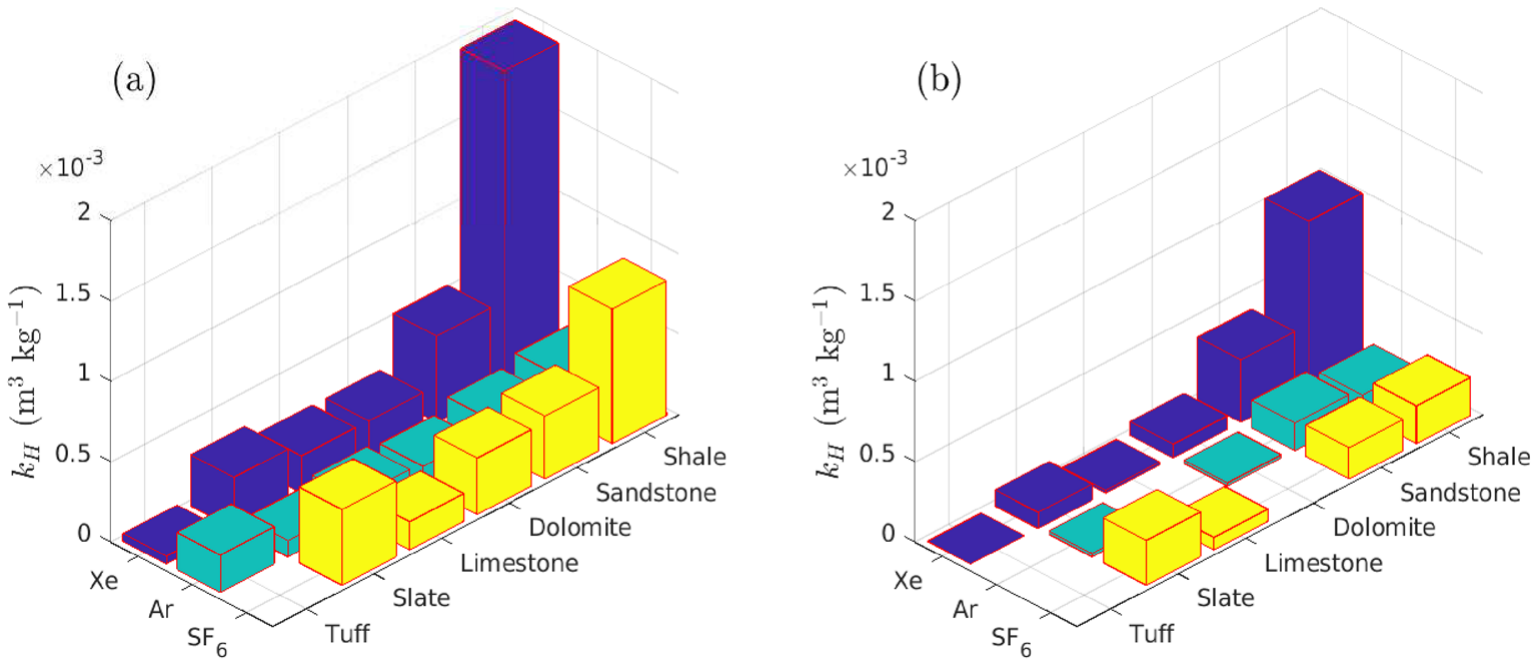
Equivalent Henry’s absorption constant of xenon, argon, and SF$_6$ in various geological materials at (**a**) 0 and (**b**) 20 $^{\circ }$C. Note that experimental data are unavailable for nonexistent bars.

The relationship between sorbed and gas concentrations at equilibrium is classified according to its curve shape that is displayed graphically by linear, concave, or convex curves^33^. The linear dependence of sorbed concentration on pore gas concentration in isotherm is described using Henry’s type absorption,

1 $$\begin{aligned} C_{\text{ H }} = k_{\text{ d }}\,c, \end{aligned}$$

where $C_{\text{ H }}$ [mol kg$^{-1}$] is Henry’s absorbed concentration, *c* [mol m$^{-3}$] is the gas concentration, and $k_{\text{ d }}$ [m$^{3}$ kg$^{-1}$] is the Henry’s absorption constant^34^. The monolayer adsorption can be expressed using the Langmuir isotherm which ignores any interaction between adsorbed molecules

2 $$\begin{aligned} C_{\text{ L }} = \frac{b'\,C'_{\text{ H }}\,c}{1+b'\,c}, \end{aligned}$$

where $b'$ [m$^3$ mol$^{-1}$] is the Langmuir affinity constant defined as the ratio of adsorption rate to desorption rate and $C'_{\text{ H }}$ [mol kg$^{-1}$] is the Langmuir capacity constant reflecting available reactive surface sites. When gas concentration is very low, $c\ll 1/b'$, the Langmuir adsorption (2) behaves as Henry’s absorption

3 $$\begin{aligned} C_{\text{ L }} \approx b'\,C'_{\text{ H }}\,c \approx k_{\text{ H }}\,c, \end{aligned}$$

where $k_{\text{ H }}$ [m$^{3}$ kg$^{-1}$] is the constant of the Langmuir linear portion. For this reason, Paul et al. ^21^ fitted sorption experimental data using Henry’s linear function (Eq. 1). The best fit of the xenon Langmuir isotherm as shown in Fig. 2 indicates that Langmuir adsorption is mainly responsible for the mass uptake while Henry’s type absorption is negligible. While the experimental data shows good agreement with the Langmuir isotherm in the full concentration range (i.e., $0 \le c \le \infty$, Fig. 2a), the sorbed concentration behaves linearly with gas concentration in the low concentration range (i.e., $0 \le c \le 7$ mol m$^{-3}$, Fig. 2b). The equivalent Henry’s constant ($k_{\text{ H }}$) is calibrated as the product of Langmuir affinity and capacity as shown in Table 1 and Fig. 3. The $k_{\text{ H }}$ value of xenon is about 40% higher than that of SF$_6$ and 3 times of the argon $k_{\text{ H }}$ value. The calibrated $k_{\text{ H }}$ reflects the combined effect Langmuir affinity (about how fast to reach isotherm) and Langmuir capacity (Eq. 3).

**Table 1:** Equivalent Henry’s absorption constant ($k_{\text{ H }}$, m$^3$ kg$^{-1}$) of xenon, argon, and SF$_6$ in various geologic materials.

Gas	T ($^{\circ }$C)	Shale	Sandstone	Slate	Dolomite	Limestone	Tuff	Mean value
Xe	0	1.97 × 10$^{-3}$	5.37 × 10$^{-4}$	2.30 × 10$^{-4}$	2.32 × 10$^{-4}$	3.18 × 10$^{-4}$	4.91 × 10$^{-5}$	5.55 × 10$^{-4}$
	20	1.03 × 10$^{-3}$	3.89 × 10$^{-4}$	8.46 × 10$^{-5}$	1.86 × 10$^{-5}$	1.01 × 10$^{-4}$	8.83 × 10$^{-7}$	2.71 × 10$^{-{\textbf {4}}}$
Ar	0	2.46 × 10$^{-4}$	1.93 × 10$^{-4}$	1.23 × 10$^{-4}$	1.47 × 10$^{-4}$	1.02 × 10 $^{-4}$	2.32 × 10$^{-4}$	1.74 × 10$^{-4}$
	20	1.26 × 10$^{-4}$	1.73 × 10$^{-4}$	1.93 × 10$^{-5}$		2.10 × 10$^{-5}$		8.47 × 10$^{-\mathbf{5}}$
SF$_6$	0	8.38 × 10$^{-4}$	3.93 × 10$^{-4}$	3.51 × 10$^{-4}$	1.75 × 10$^{-4}$	4.71 × 10$^{-4}$		4.46 × 10$^{-4}$
	20	2.35 × 10$^{-4}$	1.95 × 10$^{-4}$		7.77 × 10$^{-5}$	2.78 × 10$^{-4}$		1.96 × 10^**−4**^

Because the specific surface area and diffusivity of crushed rock samples^21^ are significantly greater than intact (core) samples^20^, the calibrated $k_{\text{ H }}$ values in Table 1 represent an upper bound of sorption capacities relevant to fracture-flow models. Additionally, most if not all sorption experiments have been performed to estimate maximum sorption capacity on mineral surfaces by preheating samples in a vacuum prior to sorption measurements. Porous and fractured geologic media in a UNE containment zone would not seem to be typically subject to similar conditions, and such pretreatment of laboratory samples would be expected to further contribute to an upper bound on the impact of sorption of a tracer-gas flow through a fracture-dominated UNE containment zone.

### Gas phase equilibrium

The phase-equilibrium partitioning of a gas component in adjoining gas and liquid phases is described as the ratio of its mole fractions in the gas phase, *y*, to its mole fraction in the liquid phase, *x*. A gas solubility model^17–24^ is applied to the gas phase-equilibrium relations for xenon and argon^36,37^ such that

4 $$\begin{aligned} K_{\text{ eq }} = \frac{y}{x} = \frac{p_{\text{ g,r }}}{S\,p_{\text{ g }}}, \end{aligned}$$

where *S* is the mole fraction (solubility) of gas component xenon and argon dissolved in the liquid phase measured at the reference gas pressure, $p_{\text{ g,r }}$, and $p_{\text{ g }}$ is the gas-phase pressure. The solubility is determined using experimental data as a function of temperature^38^

5 $$\begin{aligned} S = \exp \left[ A + \frac{B}{T_n}+C \ln T_n +F\,T_n\right] , \end{aligned}$$

where *A*, *B*, *C*, and *F* are model parameters, and $T_n$ is the temperature in Kelvin degree divided by 100. The temperature dependent $K_{\text{ eq }}$ of xenon and argon has been coupled with models of multiphase reactive transport (e.g.,^53–37^).

**Figure 4:**
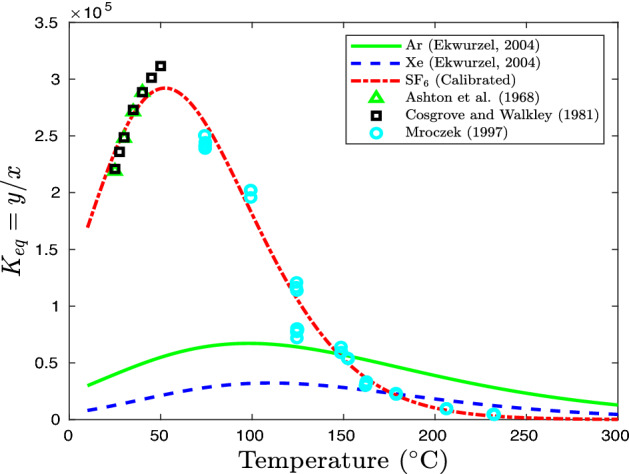
Phase equilibrium coefficient of argon, xenon, and SF$_6$ as a function of temperature. Experimental data are referred to Ashton et al.^25^, Cosgrove and Walkley^26^ and Mroczek^28^.

The solubility of SF$_6$ in water has been measured and determined as a function of temperature^25–28^. The experimental data of Ashton et al.^25^, Cosgrove and Walkley^26^, and Mroczek^28^ are used to calibrate the solubility model (5). The modeled phase-equilibrium constant is compared against experimental data as shown in Fig. 4 and the calibrated solubility constants are given in Table 2. The minimum solubility of SF$_6$, $S_{\text{ min }} = 1/K_{\text{ eq, } \text{ max }}$, is considered as the lower bound of dissolution for all gas components in uncertainty quantification.

**Table 2:** Sts that fit Eq. (5).

Gas	*A*	*B*	*C*	*F*	Reference
He	−41.4611	42.5962	14.0094		Clever^23^
Ne	−52.8573	61.0494	18.9157		Clever^23^
Ar	−57.6661	74.7627	20.1398		Clever^24^
Kr	−66.9928	91.0166	24.2207		Clever^40^
Xe	−74.7398	105.210	27.4664		Clever^40^
SF$_6$	−126.315	170.186	50.9080	0.4190	Ashton et al.^25^
					Cosgrove and Walkley^26^
					Mroczek^28^

### Radioactive decay and ingrowth

**Figure 5:**
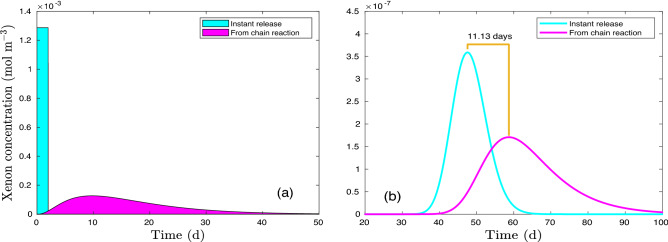
Xenon signatures are delayed by radionuclide decay. (**a**) Xenon concentration in the source area. (**b**) Xenon concentration 100 m away from source area with a flow velocity of 2 [m d$^{-1}$] and a dispersivity of 1.0 [m$^2$ d$^{-1}$].

For bounding estimates or similar approximations, isotopic xenon source terms are often conceptualized to be prompt source fluxes or impulse concentrations into the zones of subsurface or atmospheric transport^3,36^. In contrast to using simplified source terms, the Bateman equation^41^ is used in this study to describe xenon radioactivity from sequential decay and ingrowth chains (e.g.,^5,19,42–45^). Relevant solutions to the sequential decay chains are given by Cetnar^46^, Slodic̆ka and Baláz̆ová^47^, Yuan^48,49^, and Zhou et al.^50^. In addition, full-scale decay and ingrowth networks with branching and converging reactions^51^ were used to demonstrate the delayed signatures of xenon isotopes^1,52^. For example, the prompt source $^{133}$Xe concentration and that calculated using the full-scale decay and ingrowth (Fig. 5a,^5,53^) result in different xenon concentration profiles with one-dimensional transport (100 m away from the source). The solution to the 1D transport with time-dependent boundary condition is presented in Sun et al.^53^ and Cleary and Ungs^54^. As shown in Fig. 5b, the 11-day delay indicates the impact of the full scale decay and ingrowth on xenon signals qualitatively.

### Barometric pumping

Barometric pumping plays a role in gas transport in fractured rock due to the combined effect of oscillatory (pressure-driven) advection in fractures and diffusion in rock matrix^4^. Matrix diffusion is known to buffer gas signature strength in some cases with delays in gas arrival at the surface depending on the binary diffusivity of each gas^3,8,37^. Lowrey et al.^19,43^ studied the effect of barometric pumping on xenon-isotopic compositions in a single-fracture system considering both independent and chain-reaction yields from an underground nuclear detonation. Sun and Carrigan^12^ pointed out that thermally driven advection (TA) is a driving force in the early stages of gas transport following a UNE, while the role of barometric pumping increases in the post-boiling stage. Sun et al.^52^ developed a deterministic model of non-isothermal multiphase transport coupled with full-chain reactions (independent and chain yields) and qualitatively demonstrated the effect of TA on xenon isotopic concentrations. In the nearly isothermal NGME, TA is anticipated to be present as only a weak transport process due to the presence of a geothermal gradient of about 25 $^{\circ }$C km$^{-1}$ and is included along with diffusion as a base case for comparison with other driving and retarding mechanisms that we consider.

### Overpressure by air injection

Even though no high temperatures characterize the NGME, the artificial pressurization of the cavity by injection of air can be considered as an approximation of over pressuring by thermal energy following a UNE. The residual heat energy of a UNE is reflected in a temperature distribution in the chimney and surrounding area. The water phase change caused by over-boiling temperature in the chimney increases the local pressure that further drives gases away from the chimney through fractures while the capillary pressure in rock matrix drives a liquid-phase flux back to the hot chimney. The geological heat pipe in a fractured matrix system (i.e., heat transport involving change of phase of water present in the rubble-filled post-detonation cavity) keeps the local pressure in the chimney higher than its surrounding area for some time after the detonation. The physical processes driven by residual heat that results in overpressure and gas advection have already been described in high-fidelity models (e.g.,^37^). Whether thermally induced by a UNE or injection-induced as occurred during the NGME, overpressure serves as a direct driving force of gas transport between the radionuclide production point and ground surface. In simulations presented in this paper, we only consider the case of overpressure caused by air injection which is relevant to the NGME.

## Results and analyses

In addition to deterministic simulations of the NGME presented in the next subsection, both a Sobol’ sensitivity study and also probability-based simulations of surface-gas concentrations were performed. The Sobol’ sensitivity^29,30^ evaluation is carried out for identifying physical processes that have a significant influence on gas concentrations at the ground surface and for providing practical insights into which process contributes the most to the variability of gas signals. The first-order decay of a single-gas species is assumed to be deterministic since the impact on concentration profiles can be corrected using its half-life. 3600 sample points are generated using a Latin hypercube approach^55^ applied in the four-dimensional parameter space, representing gas sorption, dissolution, overpressure of the source, and barometric pumping. A normalized scale between 0 and 1 is used to describe uncertainties of gas sorption and phase equilibrium. Zero in the sorption dimension denotes $k_{\text{ H }}=0$ while 1 represents the $k_{\text{ H }}$ value (in bold) in Table 1 calibrated using experimental data, which tend to maximize the effect of sorption as discussed earlier. Zero of a gas species in the dissolution dimension represents the curve of $K_{\text{ eq }}=y/x$ scaled to the maximum value (2.95$\times$10$^5$) while one denotes its original curve (Fig. 4). Normalized scale 0 and 1 in the overpressure dimension represents ambient pressure at the source and the overpressure induced by air injection in the NGME. The range of overpressure represents the uncertainty of injection induced pressure during the NGME. The uncertainty of barometric pumping is also parameterized within 0 and 1 representing, respectively, a constant pressure (mean value of measured pressure) and recorded fluctuating atmospheric pressure obtained by Smart Samplers at the Barnwell site.

### Simulation results of deterministic models

Deterministic (NUFT) models of non-isothermal and multiphase transport^31,32^ were developed with physical processes defined in Table 3 for estimating the contributions of each process to concentration profiles. Because of uncertainties associated with containment regime transport at the Barnwell site (e.g., fracture network extent, fracture apertures and fracture density, etc.) as well as uncertainties in the injection and sampling protocols used, no attempt has been made to exactly match observations presented in Olsen et al.^15^. Rather, our dual-permeability NUFT models are based upon known or inferred properties involving depth of detonation, cavity size, fracture aperture, fracture density, and bulk permeability^1^, period between initial injection and pressurization of cavity, atmospheric pressure history, etc. A reference model (#0) with zero sorption, the lower bound of dissolution ($K_{\text{ eq }}$=2.95$\times$10$^5$), zero overpressure (representing the ambient diffusive geothermal and convective hydrological condition in the cavity), and the mean value of barometric pressure (on the ground surface) is used to compare models with a single process (#1$\sim$4), combined delaying processes (#5), combined driving processes (#6), and the full model with the full strength of 4 uncertain processes (#7). Diffusion and geothermally induced two-phase convection together are assumed to be basic and certain processes in all models.

**Table 3:** Deterministic models with multiple physical processes (including basic processes of  geothermal-gradient induced multiphase convection and diffusion).

Process	Basic process	Delaying process		Driving process	
	Convection+Diffusion	Sorption	Dissolution	Overpressure	Barometric
Model	(DF)	(SP)	(DS)	(OP)	pumping (BP)
**0**	☑	☐	☐	☐	☐
1	☑	☑	☐	☐	☐
2	☑	☐	☑	☐	☐
3	☑	☐	☐	☑	☐
4	☑	☐	☐	☐	☑
5	☑	☑	☑	☐	☐
6	☑	☐	☐	☑	☑
**7**	☑	☑	☑	☑	☑

**Figure 6:**
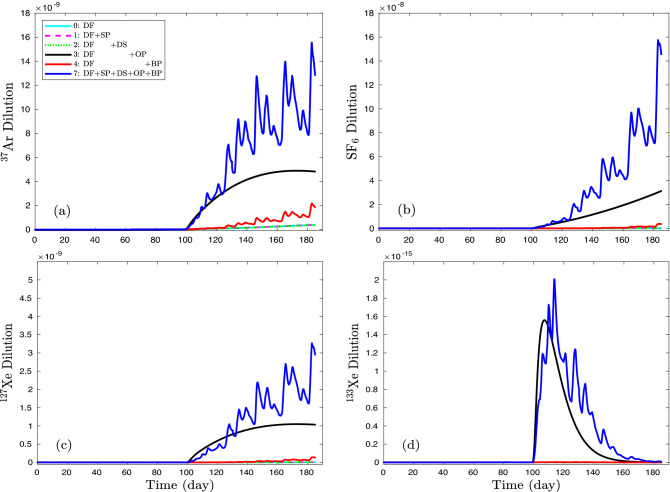
Concentration profiles are plotted for (**a**) $^{37}$Ar, (**b**) SF$_6$, (**c**) $^{127}$Xe, and (**d**) $^{133}$Xe at the depth of 0.5 m which include radioactive decay. Single delaying or driving process models (#1$\sim$4) are compared with reference models (#0 and 7). Due to the minor driving effect of diffusion and the retarding effects of sorption and dissolution, concentration curves of all gases of models #0, 1, and 2 are overlapped slightly above *x*-axis.

Figure 6 shows concentration profiles of $^{37}$Ar, SF$_6$, $^{127}$Xe, and $^{133}$Xe at the depth of 0.5 m including the effect of radioactive decay. The single-process (delaying or driving) models are compared with reference models (#0 and 7). Although it is difficult to see the offset made by sorption (dashed magenta) and dissolution (green) from the reference diffusion profile (cyan), the overpressure (black) and barometric pumping (red) concentration profiles clearly burst through the surface after 99 days. It is also observed that the sum of contributions made collectively from single-process models (#1$\sim$4) is much lower than that from the model including all processes (#7). In other words, the overpressure in the cavity and barometric pumping from the ground surface working together enhance vertical gas migration far more than the retarding processes (dissolution and sorption) reduce it.

**Figure 7:**
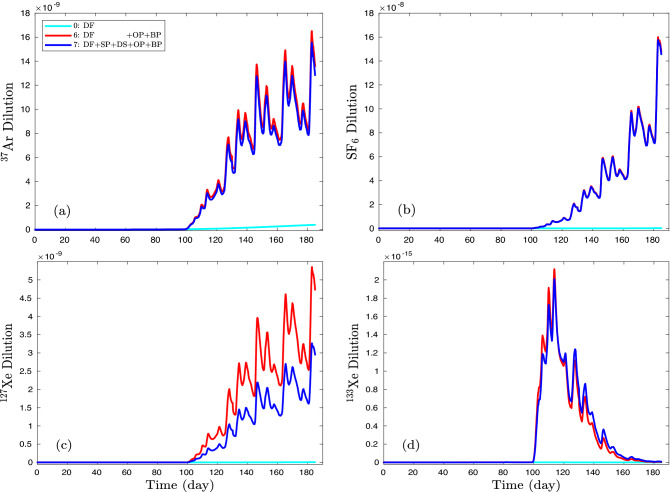
Using deterministic models, concentration profiles are plotted for (**a**) $^{37}$Ar, (**b**) SF$_6$, (**c**) $^{127}$Xe, and (**d**) $^{133}$Xe at the depth of 0.5 m. Although combined delaying or driving process models (#5, 6) are compared with reference models (#0 and 7), model #5 results are not plotted due to its negligible values.

**Figure 8:**
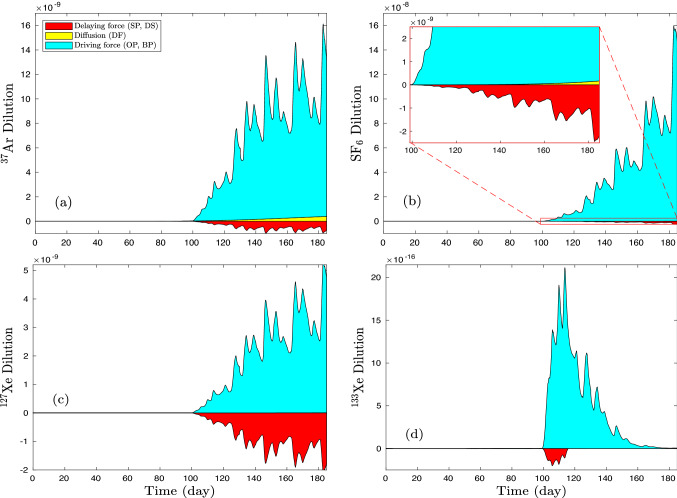
From deterministic models, the contribution of driving and retarding processes to concentration profiles is plotted for (**a**) $^{37}$Ar, (**b**) SF$_6$, (**c**) $^{127}$Xe, and (**d**) $^{133}$Xe at the depth of 0.5 m. The yellow region is the base-model (#0) profile, the red region shows the concentration difference between model #7 and model #6 indicating the effect of retarding processes (sorption and dissolution), and the cyan region shows the concentration difference between model #6 and model #0 indicating the effect of driving force (overpressure and barometric pumping).

Models #5 and 6 demonstrate the combined effect of delaying processes (sorption and dissolution) and of driving forces (overpressure and barometric pumping), respectively. Figure 7 shows the concentration profiles of models #0, 6, and 7. The minor difference between the base model (#0) and the model of double retarding effect (sorption and dissolution, #5) is not plotted. The offset between models #6 and 7 is evaluated as the effect of delaying processes (SP, DS) while the difference between models #6 and 0 is calculated as the effect of driving forces (OP, BP). The concentration profile of model #0 is the contribution of diffusion. The roles of the basic (diffusion), delaying, and driving processes are demonstrated as shown in Fig. 8. A minor role of diffusion is observed for $^{37}$Ar in Fig. 8a (yellow). Although the diffusion contribution is plotted on a log scale in the inset of Fig. 8b, it is orders of magnitude lower than that of delaying and driving processes.

Figure 6 illustrates how the two driving processes, barometric pumping (red line) and overpressure (black line), can interact in a synergistic manner transporting more gas to the surface (blue line) than either acting independently (see also Harp et al.^56^). Over pressurization can indeed drive gas to the surface along fractures. However, over pressuring of a cavity, acting alone, will also work with diffusion to drive a gas flowing upward from a region of higher concentration along a fracture as well as through the porous walls of the fracture into the rock matrix causing gradual dilution of the tracer with distance along the fracture. On the other hand, a decrease in barometric pressure, acting alone, is most effective in drawing tracer vertically near the surface. Gases are drawn by the falling pressure most effectively into the vertical fracture from the walls at shallow levels where tracer concentrations tend to be much lower than at deeper levels. However, combining both transport mechanisms tends to offset the impediments to transport of each operating alone. A vertical upward flow due to pressurization carries tracer which is gradually lost into the matrix as the tracer moves upward. Continuing this process loads the matrix with tracer. During falling atmospheric pressure, the pressure front propagates from the surface down the fracture lowering the pressure locally which not only enhances upward flow in the fracture but, more importantly, draws out tracer stored in the fracture walls into the upward flow. This explanation is consistent with the observations of time-dependent tracer concentrations obtained during and after the 10-day pressurization period while Freon was injected into the rubblized cavity in the Barnwell site experiment (Fig. 2,^1^). During pressurization, tracer levels at the surface peaked during periods of falling atmospheric pressure. When over pressurization was stopped, Freon concentration peaks gradually, not immediately, fell between October 2 and 4 from the maximum value achieved during injection, which is also consistent with the gradual depletion by barometric pumping of the near-surface storage of Freon originally loaded into the shallow rock matrix during the period of pressurization.

Our simulations further show how the four gases considered in Fig. 7 are affected to varying degrees by the retarding effects of sorption and dissolution into groundwater. For SF$_6$, the case including retarding effects (blue) almost perfectly tracks the case where retarding effects are ignored (red). The $^{37}$Ar simulations with and without retardation effects match each other reasonably well suggesting that dissolution and sorption might be expected to exert relatively minor effects on the Ar isotope at the NGME. Our simulations for $^{37}$Ar and SF$_6$ bear significant transport similarities with most of the difference in concentrations being due to the radioactive decay of the Ar isotope, which is consistent with the conclusion made by Johnson et al.^57^ concerning the tracking of Ar migration by SF$_6$. It is not surprising that the greater adsorption and dissolution associated with Xe give rise to the greatest difference between simulations with and without retardation. However, we find the decay-corrected difference between $^{127}$Xe and SF$_6$ is closer to a factor of two falling far short of the factor of ten difference in concentrations observed by Olsen et al.^15^ in the NGME. We suggest possible causes for this discrepancy in the Discussion and Conclusions section.

While $^{133}$Xe was not used in the Barnwell experiment because of its short half-life of 5.24 days relative to the anticipated several-month length of the field experiment, it is of significantly greater interest for UNE monitoring purposes than $^{127}$Xe and we ran an additional deterministic simulation to investigate how transport might affect the $^{133}$Xe signature at the surface. Unlike the $^{127}$Xe simulation with its significant difference between the cases with and without retardation, the $^{133}$Xe with and without retardation cases were quite similar in Fig. 7 suggesting that retardation effects on transport in the Barnwell experiment that are associated with a difference in half-lives between $^{133}$Xe and $^{127}$Xe are somewhat greater than the combined effects of sorption and dissolution. We then used SF$_6$ and $^{127}$Xe surface concentrations to directly estimate the time-dependent concentration of $^{133}$Xe at the surface by only correcting surface values by a factor compensating for differences in radioactive decay. The estimates of the simulated $^{133}$Xe concentration were within a factor of 2 with, surprisingly, the SF$_6$ always providing the closer match to $^{133}$Xe while $^{127}$Xe consistently underestimated the surface concentration of $^{133}$Xe. If diffusion plays little to no role in transport as appears to be the case for the NGME simulations, then radioactive-decay-induced modifications of either along-fracture or transverse concentration gradients of the surrogate tracer can be neglected and a simple decay-correction factor applied to the surface concentration of the non-radioactive surrogate should be adequate. On the other hand, if diffusive transport plays a significant role, then the modification of local concentration gradients by radioactive decay should be considered and simply multiplying the surrogate surface concentration by a decay-correction factor may not provide a sufficient approximation.

A comparison of the impact on dilution of retarding effects versus driving effects is shown in Fig. 8. The plots suggest that saturation and sorption have the greatest impact on enhancing dilution of $^{127}$Xe relative to the other gases considered. Peak reduction of dilution by delaying effects is about 40% of the maximum of driving effects compared to only about 5% of the maximum for $^{133}$Xe. For comparison, retarding effects reduce dilution of SF$_6$ by less than 1%. Figs. 6–8 raise some interesting questions regarding the use of radioactive isotopes as surrogates or substitutes for other radioactive isotopes which we consider further in the Discussion and Conclusions.

### Probability density functions of relative concentrations

**Figure 9:**
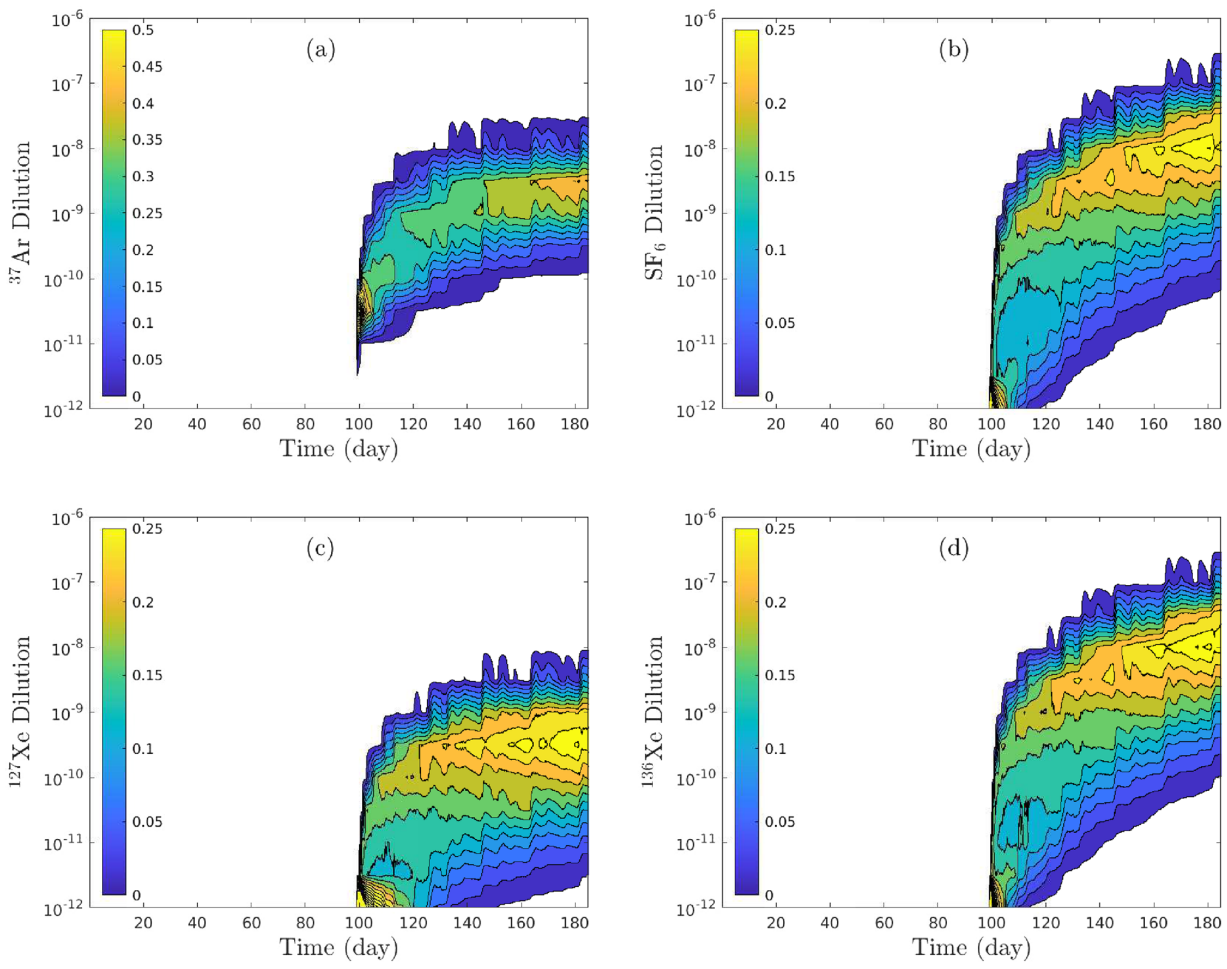
Probability densities of (**a**) $^{37}$Ar, (**b**) SF$_6$, (**c**) $^{127}$Xe, and (**d**) $^{136}$Xe relative concentrations at ground surface of Barnwell system.

The uncertain inputs (sorption, dissolution, overpressure, and barometric pumping) are assumed to be uniformly distributed in the 4-dimensional space. The probability density functions (PDFs) of $^{37}$Ar, SF$_6$,$^{127}$Xe, and $^{136}$Xe are evaluated as functions of time as shown in Fig. 9. It is observed that the probability of all four gas concentrations is negligible $(\Pr (c > 1\times 10^{-12}) \simeq 0)$ within the first 99 days (before the overpressure is applied). The PDFs of those 4 concentration profiles indicate the likelihood of detectability at the ground surface due to overpressure and barometric pumping subject to gas dissolution in partially saturated pore spaces as well as sorption on mineral surfaces.

The deterministic models of the Barnwell experiment presented earlier were intended to better understand the impacts of various combinations of driving and retarding processes. Of course, this field experiment is inherently uncertain with regard to the exact nature of the containment regime as represented by the parameters that determine transport of gases from the cavity. With the ranges of uncertainty defined previously for the parameters, we performed thousands of simulations for different gases using sets of parameters selected randomly by employing a Latin-hypercube approach^55,29^. The resulting solutions of dilution versus time were grouped into “bins” which were used to construct the PDFs. Given the uncertainty of the parameters, PDFs estimate the probability that a particular range of dilution will occur at a given time with the lighter colors (e.g., orange and yellow) representing the highest probability. A stable isotope, $^{136}$Xe, and SF$_6$ exhibit strikingly similar behaviors in the highest probability bins. Both $^{37}$Ar and $^{127}$Xe, originally injected premixed with known concentration ratios, have the highest probability of occurring at dilutions predicted to be comparable to each other which is indeed observed in the NGME^15^. However, decay correction yields comparable magnitudes to SF$_6$ which is not consistent with the field observations by Olsen et al.^15^ in the Barnwell experiment. We will speculate on the potential origins of the observed discrepancy between SF$_6$ and the radioactive tracers in the Discussion and Conclusions.

### Sobol’ sensitivity analysis

A base case model of gas transport was developed as a reference on zero sorption, minimum dissolution, zero overpressure, and mean barometric pressure. The variations of $^{37}$Ar, $^{127}$Xe, and SF$_6$ concentrations at the ground surface between 3600 models (corresponding to those 3600 sample points) and the base case model are considered as the output for uncertainty quantification

6 $$\begin{aligned} Y_{i,j} \left( t\right) = c_{i,j}\left( \mathbf{x}, t\right) - c_{i,0}\left( \mathbf{x}, t\right) \end{aligned}$$

where *Y* is the output, $i=$ [$^{37}$Ar, SF$_6$, $^{127}$Xe] is the gas species index, $j=1,2,\cdots , 3600$, is the sample index, $c_0$ and *c* are the concentration profiles of the base case model and sample-point models, $\mathbf{x}$ indicates the location (e.g., ground surface), and *t* is the time.

A Sobol’ sensitivity analysis^30,29^, which is based on decomposition of the model output variance into summands of variances of uncertain inputs, determines the contribution of each uncertain input and its interaction with other uncertain inputs to the variance of the overall model output of interest. The total sensitivity, $S_{\text{ Ti }}\left( i,t\right)$, of the output (e.g., $^{37}$Ar, $^{127}$Xe, SF$_6$ concentrations) to uncertain inputs (sorption, dissolution, overpressure, and barometric pumping represented by sample points, $j=1,2,\cdots$, 3600) is calculated as the sum of all the sensitivity indices, including all the interactive effects^58^ at all time steps. Considering the detection limit of gas components at the ground surface, the Sobol’ total sensitivity indices (TSI) are scaled using concentration profiles as

7 $$\begin{aligned} \tilde{S}_{\text{ Ti }}\left( i,~t\right) = \tilde{c}\left( t\right) \cdot S_{\text{ Ti }}\left( i,t\right) , \quad \tilde{c}\left( t\right) =\frac{\bar{c}\left( t\right) -\bar{c}_{\text{ min }}}{\bar{c}_{\text{ max }}-\bar{c}_{\text{ min }}} \end{aligned}$$

where $\tilde{c}$ is the normalized concentrations in terms of time, $\bar{c}_{\text{ min }}$ and $\bar{c}_{\text{ max }}$ are the minimum and maximum values of mean concentrations over 3600 samples.

**Figure 10:**
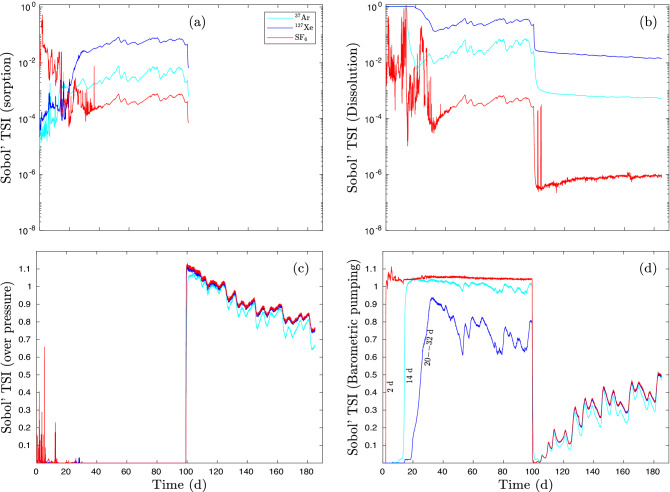
Sobol’ total sensitivity of $^{37}$Ar, $^{127}$Xe, and SF$_6$ signals at ground surface of the Barnwell system. Sobol’ total sensitivities of (**a**) sorption, (**b**) dissolution, (**c**) overpressure, and (**d**) barometric pumping.

Figure 10 shows Sobol’ total sensitivity indices $S_{\text{ Ti }}\left( i,t\right)$ of *Y* measure (6) for $^{37}$Ar, $^{127}$Xe, and SF$_6$ *Y* measures as functions of time. Although gas concentrations at the ground surface are not detected in the first 99 days as shown in Fig. 9, the Sobol’ TSI indicates that barometric pumping (Fig. 10d) dominates the variance of *Y* before the overpressure is applied at 99 days, that is, barometric pumping is always present and contributed to some upward movement of gases starting after their injection. The role of barometric pumping becomes significant at 2, 14, and 20 days, respectively according to the *Y* variances of SF$_6$, $^{37}$Ar, and $^{127}$Xe. As shown in Fig. 10b, the contribution of dissolution of SF$_6$, $^{37}$Ar, and $^{127}$Xe that retards gas transport decreases with time as barometric pumping increases its role within 2, 14, and 20 days, respectively. Gas sorption also plays a minor and negligible role before and after overpressure is applied (Fig. 10a). After overpressure takes over at 99 days (Fig. 10c), the effect of dissolution also becomes much smaller (Fig. 10b). As the role of overpressure decreases with time after 99 days, the role of barometric pumping increases. It should be noted that the effect of overpressure is eliminated only gradually after pumping is stopped since it takes time for excess pressure in the cavity as well as in the porous rock matrix between fractures to dissipate so that the transition to the dominance of barometric pumping is not instantaneous.

**Figure 11:**
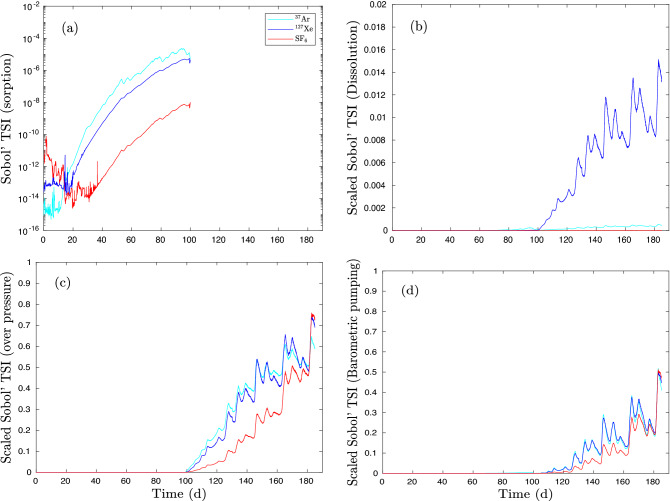
Scaled Sobol’ sensitivity of $^{37}$Ar, $^{127}$Xe, and SF$_6$ signals at ground surface of the Barnwell system. Sobol’ total sensitivities of (**a**) sorption, (**b**) dissolution, (**c**) overpressure, and (**d**) barometric pumping.

Figure 11 shows scaled Sobol’ total sensitivity indices $\tilde{S}_{\text{ Ti }}\left( i,t\right)$ of $^{37}$Ar, $^{127}$Xe, and SF$_6$ as functions of time. The scaled Sobol’ TSI can be interpreted as the contributions of four uncertain processes to measurable gas signals. Fig. 11a shows that gas sorption plays the least role in the first 99 days and has zero impact after 99 days to detectable gas signals. Fig. 11b indicates the minor role of gas dissolution after 99 days having the highest impact on transport of $^{127}$Xe. Both overpressure and barometric pumping are primarily responsible for the initial arrival of tracers at the surface.

## Discussion and conclusions

Physical processes such as driving forces (e.g., diffusion, barometric pumping, advection driven by overpressure), and retarding forces (e.g., gas sorption and dissolution) that may possibly be involved in gas migration from both field experiments and UNEs to the ground surface were investigated as separate and even more importantly as interactive processes affecting transport in the context of deterministic simulations. Considering only processes acting in isolation can lead to significant misunderstanding regarding the overall role of either a driving or retarding process. For example, gas diffusion alone is considered a driving process. But when considered with time-dependent advection of gas along a fracture, the role of diffusion is more complicated. Whether it is helping or opposing advection then depends on the strength of transverse diffusion into fracture walls and the time-dependence of advection.

Our deterministic simulations, including all driving and retarding processes, obtained similar results to the NGME regarding transport of $^{37}$Ar and $^{127}$Xe that produced concentration ratios at the surface similar to the premixed and injected values. From our simulations, we further found that a more accurate prediction of $^{133}$Xe resulted from using the concentration of SF$_6$ tracer rather than the $^{127}$Xe as a basis. Our deterministic and probabilistic simulations, when decay corrected, produced results indicating comparable magnitudes for the three gases which were not realized in the NGME in which the SF$_6$ tracer produced surface concentrations approaching 10-times those of $^{127}$Xe. The observed excess of SF$_6$ over the Xe tracer has raised concerns that the SF$_6$ tracer is somehow not appropriate for this application^57^ due to hypothesized strong mineral adsorption of Xe^21^ or unknown effects. However, the Sobol’ sensitivity study presented here does not justify a model of adsorption or dissolution strongly retarding $^{127}$Xe concentration at Barnwell even using laboratory data that is likely to over-estimate sorption effects. Comparison of the Xe and Ar observations of NGME at Barnwell (see Fig. 5 of^15^) also does not seem to support the view that delaying effects affecting xenon transport are particularly large. According to laboratory measurements, Xe is subject to greater adsorption (Fig. 3) and dissolution (Fig. 4) than Ar. Yet measured concentrations at the surface of Xe are similar to or even larger than those of Ar according to Olsen et al.^15^ indicating little apparent effect of sorption and dissolution delaying processes. The Sobol’ analysis quantifies the effect of these two retarding processes indicating that dissolution of Xe into a partially saturated porous regime has the largest effect on the concentration at the surface while sorption is not important.

Because diffusivity is lower for SF$_6$ and it is less affected by dissolution in porewater compared to the other gases, the transport of SF$_6$ might be expected to produce higher surface concentrations in a fracture dominated system with both barometric pumping and pressurization relative to $^{37}$Ar or $^{127}$Xe. However, the magnitude of the observed discrepancy between SF$_6$ and the other tracers in the NGME cannot easily be explained by tracer property differences alone and we suggest two possible contributors to the discrepancy between observed concentrations of SF$_6$ and the two radioactive tracers. During the  three-tracer injection into the rubblized Barnwell cavity at the beginning of the NGME, a 50/50 pre-mixed quantity of $^{37}$Ar and $^{127}$Xe was pumped into the airflow down the borehole while SF$_6$ was pumped into the same flow from a different tank with injection rate monitored separately from that of the premixed radioactive tracers. Injection rates as well as fluctuations in the injection rates are reported by Olsen et al.^15^. During the contemporaneous injection of the premixed radioactive tracers with SF$_6$, the SF$_6$ injection rate varied between 1.2 and 50.0 [L min$^{-1}$] while the premixed radiotracer injection rate fluctuated between 25.5–40.0 [ml min$^{-1}$] during the 10-hour injection period. The reported factor-of-40 variation in SF$_6$ injection rate, which occurred during the 10-hour period, can potentially produce order-of-magnitude spatial variations in ratios of tracer concentration in the rubblized cavity. Unlike mixing in an open cavity, the rubble material filling the detonation cavity would have inhibited post-injection mixing and homogenization of any nonuniform distribution of tracers present in void spaces in the debris. This hypothesis is supported by the analyses of gas samples taken over a period of more than 3 months from two drill-back holes into the Disko Elm cavity^59^. The samples indicated that $^4$He gas, released into the cavity at the time of or soon after detonation, had remained poorly mixed with other gases in the cavity having concentrations varying by as much as a factor of 20 even though mixing of gases in the rubble-filled cavity should have been strongest so soon after detonation. It is crucial that future experiments intended to monitor the relative transport of different tracers should be designed to ensure that the initial state of tracer distribution in a cavity is as uniform as possible given that the effects of initial heterogeneities in tracer spatial distribution may be inseparable from transport caused changes to the concentrations.

Comparing concentrations of different tracers, such as SF$_6$ and $^{127}$Xe, when extremely different sample volumes are required for analysis (0.5 liter for SF$_6$ compared to 2000 liters for $^{127}$Xe), is also problematic. It cannot be assumed that the concentration of either tracer present in a very large sample will remain constant during the subsurface gas extraction process. If the objective is to measure the effects on the concentrations of different tracers at a point on the surface or in the subsurface due to transport, what aliquot for SF$_6$ analysis taken during extraction of the 4000-times larger $^{127}$Xe sample is most representative of tracer ratios influenced by the subsurface processes discussed in this and the papers by Olsen et al.^15^ and Johnson et al.^57^? The situation becomes even more complicated recognizing that a 2000-liter sample cannot be considered a local sample even when extracted at a point in a fracture-dominated transport regime such as the Barnwell site as it may draw gases along fractures from locations potentially tens to hundreds of meters from the sampling point. Besides clouding the interpretation of the locality of a sample, a strong argument can be made that the extraction process involving large volumes can potentially distort in situ tracer concentration ratios. Equation (14) of Nilson et al.^4^ is an analytical solution describing the filtering process that removes trace gases during flow along fractures with porous walls which represents the situation encountered when extracting large samples from a fracture-dominated regime. The efficiency of the filtering process is dependent, among other parameters, on the diffusivity of each gas involved and under realistic conditions can result in differences in concentration comparable to those observed in the Barnwell site experiment according to the equation. For example, assuming a characteristic 1.5$\times$10$^{-3}$ m aperture fracture as previously estimated for the Barnwell site^1^ and a fracture wall porosity of 0.1, differential filtering during flow of gases at the rate of 0.01 [m s$^{-1}$] will result in $^{127}$Xe concentration falling to about 1/10 that of SF$_6$ over a fracture length of 50 m. While representing a plausible explanation for fully explaining the difference in observed concentrations between SF$_6$ and $^{127}$Xe for the appropriate set of fracture parameters, it would also be expected to cause a significant enrichment in concentrations of $^{127}$Xe relative to $^{37}$Ar while only a smaller enrichment is generally seen according to Fig. 5 of Olsen et al.^15^. In any case, the differential filtering effect should be considered for its potential ability to distort gas ratios during extraction of a very large gas sample, but we conclude that it is at best only a partial explanation for the disparity observed between SF$_6$ and $^{127}$Xe concentrations by Olsen et al.^15^. Given the large fluctuations in injection rate of SF$_6$ reported by Olsen et al.^15^ along with observations of a non-uniform cavity gas distribution persisting for months following the Disko Elm event^59^, it seems likely that the main reason for the excess of observed SF$_6$ is the production of an initial non-uniform spatial distribution in the debris-filled cavity that is then channeled into the overlying fracture system.

In conclusion, we have presented a variety of simulations representing a re-analysis of the scenario posed by the NGME Barnwell injection experiment reported by Olsen et al.^15^ involving both radioactive and chemical tracers. The experiment, originally planned as a study of barometric pumping and its effect on different tracers, was modified after no signal was observed at the surface by re-pressurizing the detonation cavity at 99 days following initial injection of tracers. Our simulations consider the geometry and aspects of the hydrology of the site although many uncertainties remain involving the fracture pathways to the surface and their effect on different gas compositions. We have now added the delaying effects of sorption from laboratory studies to our models but found with the aid of both deterministic simulations and a sensitivity study that they were not significant in this particular case. Finally, if the objective of a field experiment is only to simulate what might be observed during an onsite inspection of a potential unannounced nuclear test site, then performing large-volume extraction sampling, as in the NGME, is a reasonable approach as it includes the contribution to the subsurface flow regime associated with the necessary large-volume extraction currently required for radiologic analysis. However, if the objective is to observe how gas migrates to the surface following a UNE with the assurance that the gas composition of a sample is actually characteristic of the sampling location, then small volume sampling involving non-radioactive tracers would seem to be the preferred approach. Finally, this study was primarily intended to evaluate gas transport processes specific to the NGME as well as provide insight into the origin of delayed signatures from UNEs. To properly quantify the influence of these processes on UNE gas signatures, future modeling must take into account the dynamics and conditions of the early time post-detonation environment which are not considered here.

## Supplementary Information

Supplementary Information.
