# Supplementary material for: Evaluation of subsurface transport processes of delayed gas signatures applicable to underground nuclear explosions
Source: Sci Rep. 2022 Aug 1;12:13169. doi: 10.1038/s41598-022-16918-5 (PMC9343667; doi:10.1038/s41598-022-16918-5)
Supplement: Supplementary file 1 — Supplementary Information. [file 41598_2022_16918_MOESM1_ESM.pdf]

Supplementary Information for
Evaluation of subsurface transport processes of delayed gas signatures applicable to underground nuclear explosions

Charles R. Carrigan, Yunwei Sun[1], Tarabay Antoun
Lawrence Livermore National Laboratory, Livermore, CA, USA.

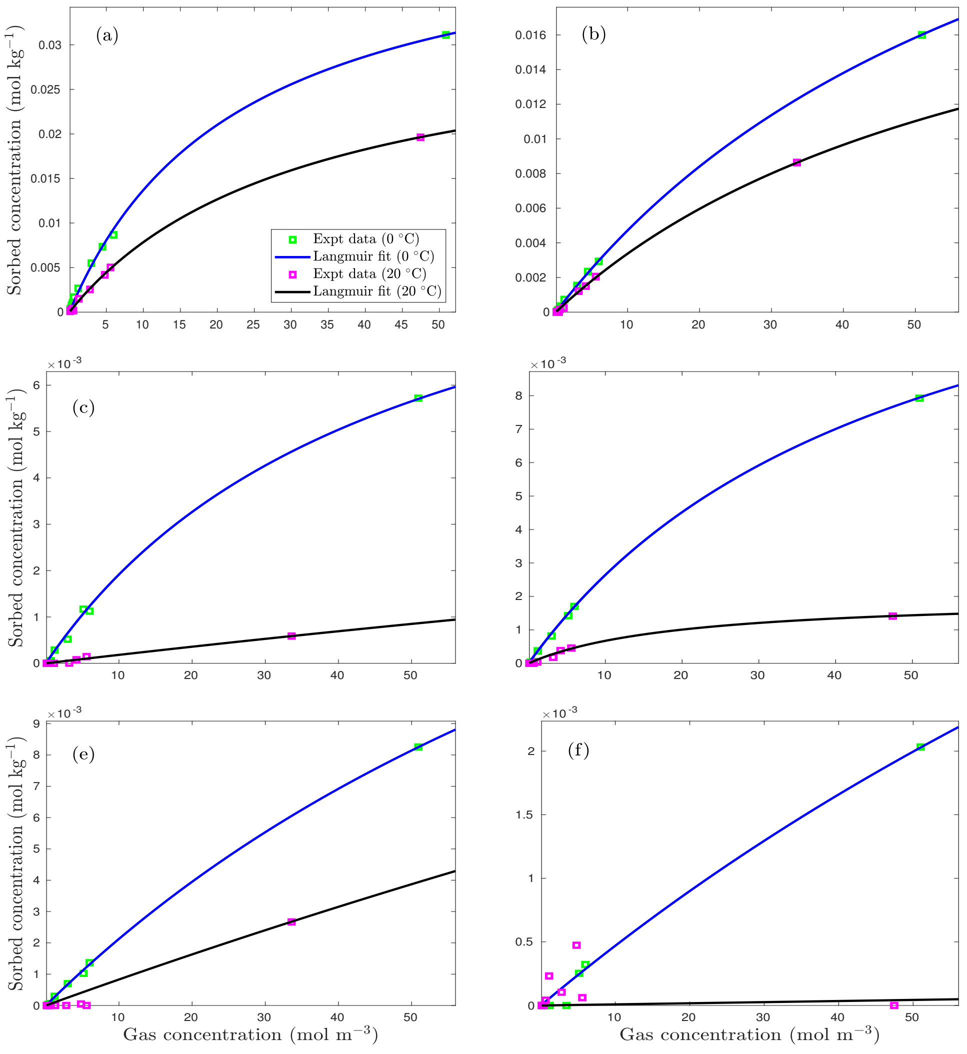

Figure S1: Xenon sorption in (a) shale (b) sandstone (c) dolomite (d) limestone (e) slate and (f) tuff calibrated using experimental data (Paul et al., 2018) and Langmuir isotherm (Eq. 2). Blue and black curves represent the best fit at 0 and 20 °C to experimental data, shown as green and magenta squares for the two temperatures, respectively.

[1]Corresponding author. *E-mail addresses*: sun4@llnl.gov (Y. Sun)
IM release number: LLNL-JRNL-827136
